# Access to hip and knee replacement surgery in patients with chronic diseases according to patient-reported pain and functional status

**DOI:** 10.1186/s12913-020-05464-3

**Published:** 2020-07-01

**Authors:** Bélène Podmore, Andrew Hutchings, Sujith Konan, John Robson, Jan van der Meulen

**Affiliations:** 1 Department of Health Services Research & Policy, London School of Hygiene & Tropical Medicine, 15-17 Tavistock Place, London, WC1H 9SH UK; 2 Clinical Effectiveness Unit, The Royal College of Surgeons of England, London, UK; 3 Consultant Orthopaedic Surgeon, University College London Hospitals NHS Foundation Trust, London, UK; 4 Centre for Primary Care and Public Health, Queen Mary University of London, London, UK

**Keywords:** Healthcare disparities, Access to surgery, Chronic disease, Surgical procedure

## Abstract

**Background:**

An increasing number of patients undergoing hip or knee replacement have chronic diseases. It has been suggested that the presence of chronic diseases may affect access to this type of surgery in the English National Health Service (NHS). We examined the access to hip and knee replacement surgery in patients with and without chronic diseases according to preoperative patient-reported pain, functional status and symptom duration.

**Methods:**

We analysed data of 640,832 patients who had hip or knee surgery between 2009 and 2016 in England. Multivariable regression was used to estimate the impact of 11 chronic diseases on severity of joint problems as measured on a scale from 0 to 48 by Oxford Hip (OHS) and Knee Scores (OKS) just before surgery and on likelihood of long-standing joint problems (> 5 years pre-operatively).

**Results:**

Patients with chronic diseases reported more severe joint problems than patients without (OHS differences ranged from 1.1 [95% CI 0.93, 1.2] to 2.5 [95% CI 2.3, 2.7] and OKS differences from 0.5 [95% CI 0.3, 0.7] to 2.6 [95% CI 2.4, 2.7] for the 11 chronic diseases) but the differences remain small. When analysed separately, patients with chronic diseases reported both more severe pain and poorer functional status. Six chronic diseases in hip patients and two in knee patients increased the likelihood that they had long-standing joint problems. The severity of joint problems just before surgery increased with the number of chronic diseases (OHS differences; one chronic disease (1.5 [95% CI 1.4, 1.5]) to four or more (5.8 [95% CI 5.6, 6.0])).

**Conclusions:**

Patients with chronic diseases reported more severe joint problems immediately before hip or knee replacement surgery suggesting they have hip or knee replacement later in the course of their joint disease.

## Background

Hip and knee replacement surgery is one of the most common and effective surgical treatments leading to significant improvements in quality of life [1]. Despite this in publicly funded healthcare systems such as England [2], Canada [3], and New Zealand [4] eligibility criteria restricting access to hip and knee replacement surgery, have recently been introduced to limit inappropriate use of joint replacement surgery and reduce healthcare cost. Eligibility criteria in England have included the severity of preoperative functional status [5] and pain [2], the requirement that a patient’s body mass index is lower than 30 kg/m^2^, and the optimisation of pre-existing chronic diseases [6–8]. There is no evidence, however, to suggest that limiting access according to any of these criteria is justified and these policies are not supported by clinical guidelines issued by the National Institute for Health and Care Excellence (NICE) [9]. Furthermore, in a comprehensive systematic review of 70 studies, on outcomes of joint replacement surgery comparing patients with and without chronic diseases, the evidence does not suggest patients with chronic diseases benefit less from hip and knee replacement surgery [10].

In a recent qualitative study, we explored the views of healthcare professionals in the English NHS about referring and selecting patients with chronic diseases for joint replacement surgery [11]. These professionals reported that some patients with chronic diseases are not ‘prepared’ for surgery because their chronic diseases are not adequately controlled. As a result, these patients are often sent back to their general practitioner in primary care, fragmenting and delaying the surgical management of their joint problems, in some cases preventing surgery altogether [12]. In addition, such delays could lead to increased functional deterioration and pain of the osteoarthritic hip and knees and thereby also increased costs. Advanced osteoarthritis of the hip and knee is associated with increased health service use and opioid use [13, 14].

Previous research investigating variation in access to joint replacement surgery has used two different approaches. Some papers have measured access indirectly from a population perspective by focusing on those not receiving surgery and seeking to measure unmet need [15]. Others have looked at those who did receive surgery, studying variation in utilisation of surgery according to factors such as geographical area [16] or socioeconomic status [17]. The Patient Reported Outcome Measures (PROMs) programme that is being carried out in the English National Health Service (NHS) has provided a new opportunity to explore access as it provides information on the severity and the duration of the joint problems just before surgery in a nationally representative sample [17]. If there were differences in access, we might expect to see differences in the severity of joint problems and in their duration according to the presence of chronic disease. A similar approach has been used previously to look at the impact of socioeconomic status [17, 18].

In this paper, we therefore examined associations of the severity of joint problems (overall and separately in terms of pain and functional status) and the duration of the joint problems in patients with different chronic diseases just before hip or knee replacement surgery to get a better understanding of the impact that chronic diseases have on access to joint replacement surgery.

## Methods

### Data sources

**Fig. 1:**
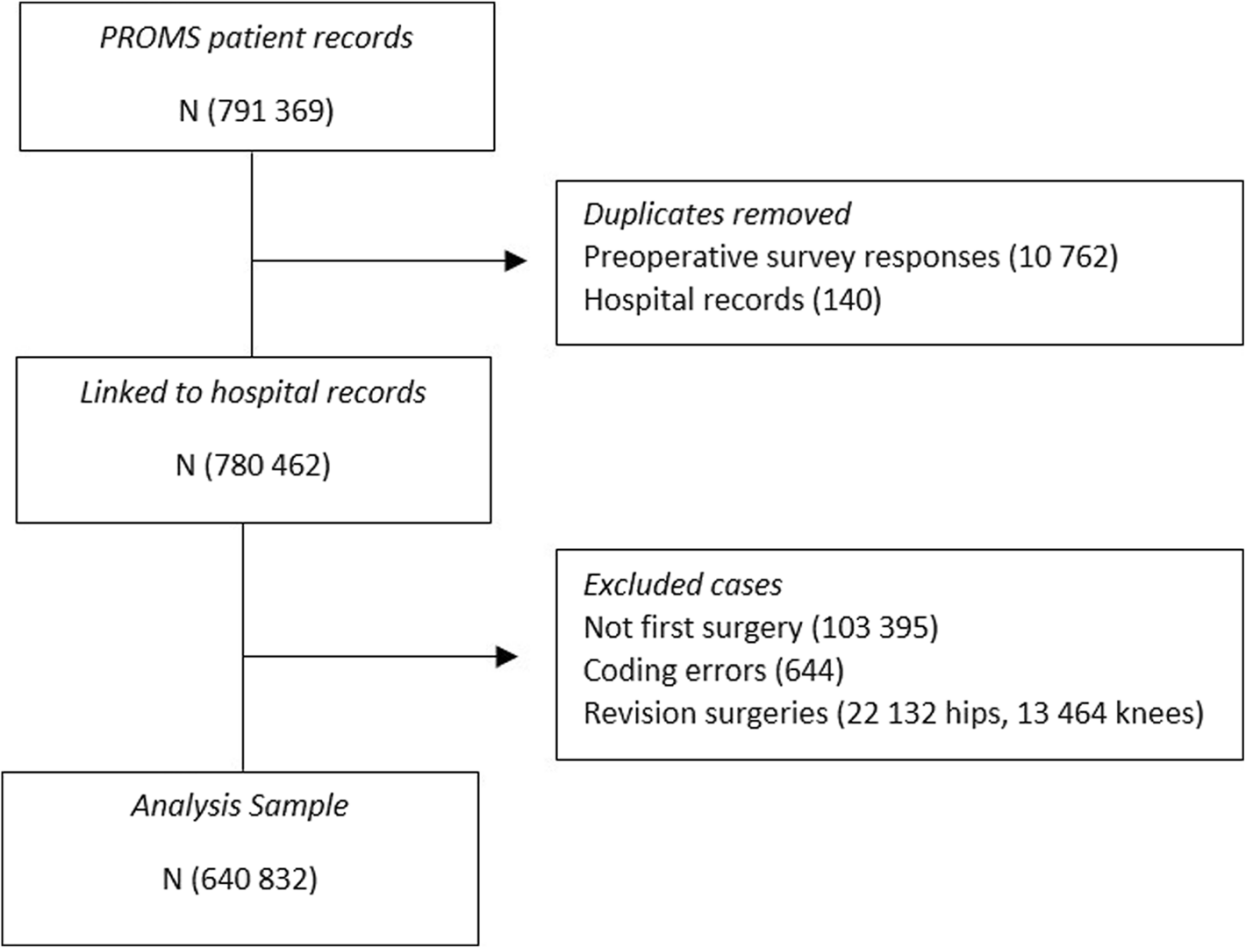
Data Flow Chart

We used data from the English national PROMs programme for elective hip and knee replacement surgery [19]. All NHS providers are required to participate and patients are asked to report their joint problems and wellbeing at the preoperative assessment clinic or on admission to hospital and then again 6 months after surgery. Over 75% of eligible patients complete the preoperative questionnaire [20]. The PROMs data were linked at patient level to data from the Hospital Episode Statistics (HES) database. HES contains administrative records of all admissions to all NHS hospitals in England. Eligibility was restricted to the first primary replacement surgery (Fig. 1).

### Defining chronic diseases

The 11 chronic diseases that were included in the analysis were defined using ICD-10 codes in the linked hospital admissions HES data up to 1 year prior to the surgery. The 11 chronic diseases comprised heart disease, high blood pressure, problems caused by a stroke, leg pain when walking due to poor circulation, lung disease, diabetes, kidney disease, nervous system disease, liver disease, cancer, and depression. These chronic disease categories, used in the PROMs questionnaire and based on the work of Bayliss et al. [21], were selected because it allowed using a combination of already existing ICD-10 diagnosis-based comorbidity indices (Elixhauser, Royal College of Surgeons Charlson and Quan Charlson Comorbidity Index).

### Measures

We used the Oxford Hip (OHS) and Knee Scores (OKS) as our measures of severity of joint problems just before surgery. These are derived from patient responses to 12 questions about pain and limits on physical functioning caused by the hip or the knee. Responses to each question are measured on a 5-point scale. Response values are added up to produce an overall scale from 0 (worst) to 48 (best). The OKS and OHS have been validated and found to correlate with surgeon assessment of symptoms [22].

We also considered the questions related to pain and those related to functional status in the OHS and OKS separately. The severity of joint problems is typically measured using disease-specific measures such as the OKS and OHS. The challenge of using these measures, which are designed to assess only the severity of the hip or knee problem, is that they may also be influenced by chronic diseases [23, 24]. To explore this further, we looked separately at the scores for pain (for example, night pain and sudden pain) and for functional status (for example, ability to go shopping on your own and climb a flight of stairs). We hypothesised that pain is more ‘joint-specific’ than functional status and that it is therefore less likely to be influenced by chronic diseases. This approach has been used before to study the impact of pain and of functional status on patient satisfaction after hip and knee replacement surgery [25]. For the OKS, scores for the five questions on pain were added together as were those for the seven on functional status (see supplementary material). For the OHS, there were six questions each on pain and functional status.

A categorical measure of duration of joint problems was derived from responses to a single question asking patients how long they had experienced problems with their hip or the knee on which they were about to have surgery. The four response categories included ‘Less than 1 year’, ‘1–5 years’, ‘6–10 years’, and ‘More than 10 years’. We defined long-standing hip or knee problems as problems with a duration of joint problems of more than 5 years pre-operatively.

### Statistical analysis

We estimated adjusted differences in mean preoperative pain and functional status using multivariable linear regression and calculated the mean scores according to the presence or absence of each chronic disease. We also estimated odds ratios (ORs) for having long-standing hip or knee problems for each chronic disease using multivariable logistic regression. The impact of the number of chronic diseases (1, 2, 3, or ≥ 4) on the severity of joint problems and duration of joint problems was also investigated to explore the effect of having multiple chronic diseases.

We adjusted for sociodemographic factors including age, sex, ethnicity and socioeconomic status (Index of Multiple Deprivation [26]) and other chronic diseases. Hospitals were added as a random effect. Missing values for ethnicity, age, sex and socioeconomic status were imputed with chained equations. Analyses were run on each of 10 imputed data sets and estimated parameters were combined using Rubin’s rules [27]. Statistical results are presented with 95% confidence intervals and *p*-values. All statistical analysis were carried out using STATA V.15.

## Results

### Patient characteristics

Six hundred forty thousand eight hundred thirty-two patients were eligible (Table 1). Their mean age was 68 and 41.8% were male. About 3% of patients reported a minority ethnic background with Black or Black British being the largest group but there was a high percentage of missing data. The most common chronic diseases were high blood pressure (52.8%), heart disease (17.8%), and lung disease (14.5%). The least common chronic disease was liver disease (0.60%). 35.3% of patients had one chronic disease and 32.3% two or more.

**Table 1:** Population characteristics

Characteristic	Hip replacement	Knee replacement
**No. of patient**	312,079 (48.7)	328,753 (51.3)
**Mean (SD) OHS or OKS**	17.4 (8.25)	18.3 (7.87)
**Mean (SD) EQ-5D**	0.33 (0.33)	0.39 (0.32)
**Long-standing problems, n (%)**	57,827 (18.5)	141,559 (43.1)
**Age, mean (range)**	68 (18–105)	69 (18–102)
**Gender, n (%)**		
Male	126,925 (40.7)	140,971 (42.9)
Female	184,982 (59.3)	187,525 (57.0)
Missing, not stated	172	257
**Socioeconomic status by quintile group, n (%)**		
1 (least deprived)	74,380 (23.4)	69,582 (21.2)
2	76,164 (24.4)	74,799 (22.8)
3	55,793 (17.9)	62,851 (19.1)
4	52,194 (16.7)	60,177 (18.3)
5 (most deprived)	50,408 (16.2)	58,327 (17.7)
Missing	3140	3017
**Ethnicity, n (%)**		
White or White British	271,959 (98.3)	279,159 (94.5)
Mixed background	546 (0.19)	836 (0.28)
Asian or Asian British	1239 (0.45)	10,445 (3.53)
Black or Black British	1703 (0.62)	3347 (1.13)
Chinese or other ethnic	1150 (0.42)	1706 (0.58)
Missing	35,482	33,260
**Chronic disease, n (%)**		
Heart disease	53,277 (17.1)	60,755 (18.5)
High Blood pressure	151,163 (48.4)	187,815 (57.1)
Stroke	3227 (1.03)	3530 (1.07)
Leg pain due to poor circulation	5140 (1.65)	4955 (1.51)
Lung Disease	43,481 (13.9)	51,176 (15.6)
Diabetes	29,535 (9.46)	44,813 (13.6)
Kidney Disease	16,428 (5.26)	18,000 (5.48)
Diseases of the Nervous System	8483 (2.72)	9741 (2.96)
Liver Disease	1888 (0.60)	1931 (0.59)
Cancer	6354 (2.04)	5545 (1.69)
Depression	13,367 (4.28)	14,814 (4.51)
**Count of chronic diseases, n (%)**		
0	113,479 (36.4)	94,290 (28.7)
1	107,139 (34.3)	119,012 (36.2)
2	59,976 (19.2)	75,202 (22.9)
3	22,929 (7.35)	29,761 (9.05)
4+	8556 (2.74)	10,488 (3.19)

### Severity of joint problems

Patients with any of the 11 chronic diseases for both hip and knee replacement surgery reported more severe joint problems than patients without chronic diseases just before surgery (Table 2). For hip replacement surgery, adjusted differences in severity of joint problems ranged from 1.06 (95% CI 0.93, 1.19) for kidney disease to 2.49 (95% CI 2.31, 2.66) for diseases of the nervous system. For knee replacement surgery, adjusted differences in severity of joint problems ranged from 0.46 (95% CI 0.26, 0.66) for cancer patients to 2.58 (95% CI 2.42, 2.73) for patients with diseases of the nervous system. The largest differences in severity of joint problems for both hip and knee replacement were reported by patients with diseases of the nervous system, depression and liver disease and the smallest differences for high blood pressure, cancer and kidney disease.

**Table 2:** Preoperative severity of joint problems (OHS in hip replacement/OKS in knee replacement) separated by functional status and pain according to chronic diseases (adjusted according to age, sex, ethnicity, SES and other chronic diseases)

Chronic disease	Hip replacement OHS				Knee replacement OKS			
	Total (0 worse and 48 best)		Functional status (0 worst 24 best)	Pain (0 worst 24 best)	Total (0 worse and 48 best)		Functional status (0 worse and 28 best)	Pain (0 worse 20 best)
	Mean score	Adjusted difference (95% CI)	Adjusted difference (95% CI)	Adjusted difference (95% CI)	Mean score	Adjusted difference (95% CI)	Adjusted difference (95% CI)	Adjusted difference (95% CI)
**Heart disease**								
**No**	17.7	–	–	–	18.4	–	–	–
**Yes**	16.0	−1.29 (−1.37, −1.21)	−0.68 (−0.72, − 0.64)	− 0.61 (− 0.65, − 0.57)	17.6	− 1.05 (− 1.12, − 0.98)	− 0.70 (− 0.74, − 0.66)	− 0.35 (− 0.38, − 0.32)
**High blood pressure**								
**No**	18.2	–	–	–	18.9	–	–	–
**Yes**	16.5	− 1.22 (− 1.29, − 1.17)	− 0.69 (− 0.73, − 0.66)	− 0.54 (− 0.57, − 0.50)	17.8	− 0.87 (− 0.92, − 0.81)	− 0.59 (− 0.62, − 0.55)	−0.28 (− 0.31, − 0.26)
**Stroke**								
**No**	17.4	–	–	–	18.3	–	–	–
**Yes**	14.5	−1.39 (− 1.67, − 1.10)	−0.81 (− 0.96, − 0.66)	−0.57 (− 0.72, − 0.43)	16.2	−1.15 (− 1.40, − 0.89)	− 0.84 (− 1.00, − 0.68)	−0.30 (− 0.41, − 0.19)
**Leg pain due to poor circulation**								
**No**	17.4	–	–	–	18.3	–	–	–
**Yes**	15.3	−1.28 (− 1.50, − 1.06)	−0.62 (− 0.74, − 0.50)	−0.66 (− 0.78, − 0.55)	17.4	−0.83 (− 1.05, − 0.62)	−0.59 (− 0.73, − 0.46)	−0.24 (− 0.33, − 0.15)
**Lung Disease**								
**No**	17.7	–	–	–	18.6	–	–	–
**Yes**	15.6	−1.49 (− 1.57, − 1.41)	− 0.70, (− 0.75, − 0.66)	−0.79 (− 0.83, − 0.75)	16.7	− 1.21 (− 1.28, − 1.14)	− 0.78 (− 0.83, − 0.74)	−0.43 (− 0.46, − 0.40)
**Diabetes**								
**No**	17.6	–	–	–	18.5	–	–	–
**Yes**	15.7	−1.31 (− 1.41, − 1.21)	−0.72 (− 0.77, − 0.67)	−0.59 (− 0.64, − 0.53)	16.8	−1.26 (− 1.34, − 1.18)	− 0.84 (− 0.89, − 0.79)	−0.42 (− 0.45, − 0.38)
**Kidney Disease**								
**No**	17.5	–	–	–	18.3	–	–	–
**Yes**	15.3	−1.06 (− 1.19, −0.93)	−0.60 (− 0.67, − 0.53)	−0.46 (− 0.53, − 0.39)	17.1	−0.82 (− 0.94, − 0.71)	−0.57 (− 0.65, − 0.50)	−0.25 (− 0.30, − 0.20)
**Diseases of the Nervous System**								
**No**	17.5	–	–	–	18.4	–	–	–
**Yes**	14.4	−2.49 (− 2.66, − 2.31)	− 1.48 (− 1.57, − 1.39)	−1.01 (− 1.10, − 0.91)	15.5	−2.58 (− 2.73, − 2.42)	− 1.87 (− 1.96, − 1.77)	−0.71 (− 0.78, − 0.65)
**Liver Disease**								
**No**	17.4	–	–	–	18.3	–	–	–
**Yes**	14.0	−2.29 (− 2.65, − 1.93)	−1.28 (− 1.48, − 1.08)	−1.01 (− 1.20, − 0.82)	16.9	−1.30 (− 1.64, − 0.97)	− 0.90 (− 1.11, − 0.69)	−0.41 (− 0.55, − 0.26)
**Cancer**								
**No**	17.4	–	–	–	18.3	–	–	–
**Yes**	16.4	−1.22 (− 1.42, − 1.03)	−0.72 (− 0.83, − 0.61)	−0.50 (− 0.61, − 0.40)	18.6	−0.46 (− 0.66, − 0.26)	−0.35 (− 0.48, − 0.22)	−0.11 (− 0.20, − 0.03)
**Depression**								
**No**	17.5	–	–	–	18.4	–	–	–
**Yes**	14.6	−2.07 (− 2.21, − 1.93)	−1.12 (− 1.19, − 1.04)	−0.95 (− 1.03, − 0.88)	15.3	−1.98 (− 2.10, − 1.85)	−1.31 (− 1.38, − 1.23)	− 0.67 (− 0.73, − 0.62)

When looking at pain and functional status scores separately, we found that patients with chronic diseases reported not only worse functional status but also more pain just before surgery than patients without chronic diseases for each of the 11 chronic diseases (Table 2). Similar to the overall OHS and OKS score, the stronger impact on both pain and functional status scores was found in patients with diseases of the nervous system and depression and the lowest in patients with kidney disease and cancer.

### Long-standing joint problems

**Fig. 2:**
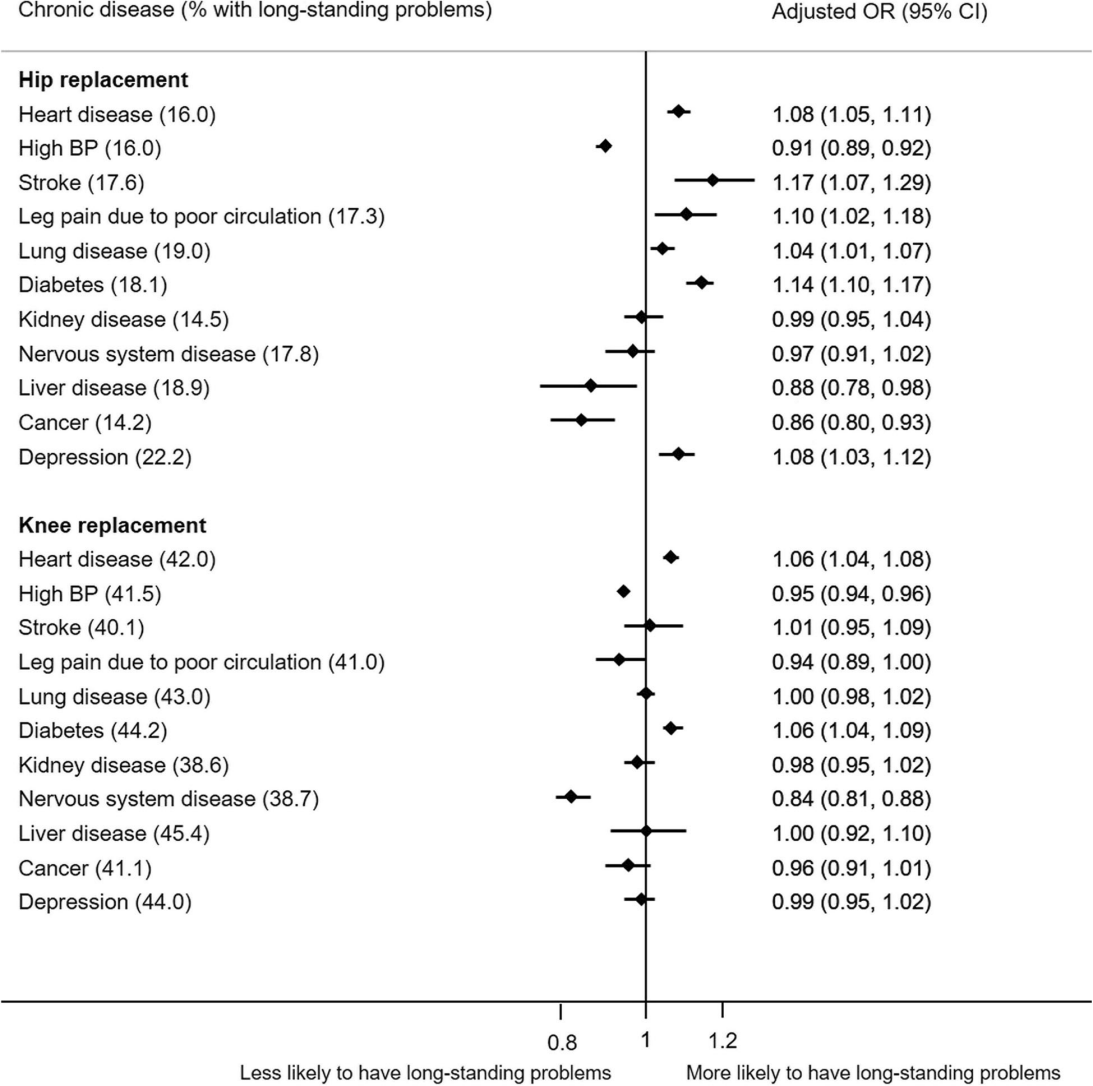
Impact of chronic diseases on long-standing joint problems (duration> 5 years pre-operatively) (95% CI) (adjusted according to age, sex, ethnicity, SES and other chronic disease)

18.5% of patients who had hip replacements reported long-standing joint problems (> 5 years) and 43.1% of those who had knee replacements. The impact of different chronic diseases on the likelihood that patients reported long-standing hip or knee problems showed a mixed picture (Fig. 2). For hip replacement surgery, six chronic diseases increased the likelihood that patients reported long-standing problems whereas three others reduced it. For knee replacement, the likelihood that patient reported long-standing problems was increased by two chronic diseases and decreased by two others.

### Multiple chronic diseases

The severity of joint problems just before surgery increased with the number of chronic diseases (Table 3). Compared with patients without chronic diseases who had a hip replacement, the adjusted differences in OHS increased from 1.45 (95% CI 1.38, 1.52) for patients with one chronic disease to 2.79 (95% CI 2.70, 2.87) for patients with two chronic diseases. The adjusted differences were largest for patients with four or more chronic diseases (5.79, 95% CI 5.61, 5.96). Compared with patients without chronic diseases who had a knee replacement, the adjusted differences increased from 1.06 (95% CI 0.99, 1.12) for patients with one chronic disease to 4.79 (95% CI 4.64, 4.94) for patients with four or more chronic diseases. We observed a similar gradient both in hip and in knee replacement surgery patients when looking at pain and functional status separately. In patients with four chronic diseases, irrespective of whether they had a hip or a knee replacement, the most common combination was high blood pressure, heart disease, diabetes, and lung disease.

**Table 3:** Preoperative severity of joint problems (OHS in hip replacement and OKS in knee replacement) and likelihood of long-standing problems by the number of chronic diseases (95% CI, *P*-value for trend) (adjusted according to age, sex, ethnicity and SES)

Count of chronic diseases	Severity of joint problems (0 worse and 48 best)			Functional status (0 worst 24 best)			Pain (0 worst 24 best)			Long-standing joint problems (duration > 5 years pre-operatively)		
	Mean score	Adjusted difference (95% CI)	***P***-value	Mean score	Adjusted difference (95% CI)	***P***-value	Mean score	Adjusted difference (95% CI)	***P***-value	%	Adjusted OR (95% CI)	***P***-value
**Hip replacement:OHS**												
**0**	18.8	Reference		10.6	Reference		8.21	Reference		21.3	Reference	
**1**	17.4	−1.45 (−1.52, −1.38)	< 0.001	9.73	−0.79 (−0.83, − 0.76)	< 0.001	7.67	− 0.66 (− 0.69, − 0.62)	< 0.001	17.3	0.93 (0.91, 0.95)	0.076
**2**	16.2	−2.79 (− 2.87, − 2.70)		9.03	−1.52 (− 1.56, − 1.47)		7.17	−1.28 (− 1.32, − 1.23)		16.5	0.98 (0.95, 1.00)	
**3**	14.9	−4.15 (− 4.27, − 4.04)		8.31	− 2.23 (− 2.30, − 2.17)		6.58	− 1.92 (− 1.98, − 1.86)		16.1	1.02 (0.98, 1.06)	
**4+**	13.3	−5.79 (− 5.96, − 5.61)		7.42	− 3.13 (− 3.23, − 3.03)		5.89	− 2.66 (− 2.75, − 2.56)		17.5	1.15 (1.09, 1.23)	
**Knee replacement:OKS**												
**0**	19.5	Reference		12.5	Reference		7.01	Reference		45.7	Reference	
**1**	18.5	−1.06 (−1.12, −0.99)	< 0.001	11.7	−0.72 (− 0.76, − 0.68)	< 0.001	6.77	−0.34 (− 0.37, − 0.31)	< 0.001	42.2	0.95 (0.93, 0.97)	0.663
**2**	17.6	−2.16 (− 2.24, − 2.09)		11.0	−1.50 (− 1.51, − 1.42)		6.51	− 0.70 (− 0.73, − 0.67)		41.9	0.97 (0.95, 0.99)	
**3**	16.5	−3.38 (− 3.48, − 3.28)		10.3	−2.28 (− 2.34, − 2.22)		6.17	− 1.10 (− 1.15, − 1.06)		41.8	0.98 (0.96, 1.01)	
**4+**	15.1	−4.79 (− 4.94, − 4.64)		9.39	−3.21 (− 3.31, − 3.11)		5.75	− 1.58 (− 1.64, − 1.51)		41.9	0.99 (0.95, 1.04)	

The impact of the number of chronic diseases on the likelihood of reporting long-standing joint problems was inconsistent. In hip patients, only patients with four or more chronic diseases were more likely to report long-standing problems (OR 1.15, 95% CI 1.09, 1.23). In knee patients, an increasing number of chronic diseases had no impact on the likelihood of patients reporting long-standing problems.

## Discussion

This study demonstrates that compared to patients without chronic diseases, patients with chronic diseases, especially those with liver disease, depression, and diseases of the nervous system, reported more severe joint problems immediately before undergoing a hip or knee replacement. Patients with chronic diseases reported not only worse functional status but also more pain just before surgery which suggests that patients with chronic diseases have truly worse joint problems regardless of any direct impact of chronic diseases on the disease-specific measure. When looking at the number of chronic diseases, the severity of the joint problems increased with the number of chronic diseases. Patients with chronic diseases however reported little to no difference in duration of their joint problems compared to patients without chronic diseases although the difference was larger in patients undergoing hip replacement compared to patients undergoing knee replacement.

The observed differences in severity of joint problems were small but statistically significant for all of the 11 different chronic diseases. To interpret the size of the difference, they can be compared with defined ‘minimally important differences’ (MID), the smallest important differences in scores that patients report as beneficial. Suggested MID values are five points for both the OHS and OKS [28]. Only hip and knee patients with four or more chronic diseases reported differences in OHS and OKS scores more than five points larger than patients without chronic diseases.

Our findings about the duration of joint problems were not in alignment with the findings related to the severity of joint problems just before surgery. These inconsistent results may be due to recall bias, because patients may find it difficult to remember the actual onset of their hip or knee problems. Patients were asked, ‘How long they had experienced problems with the hip or the knee on which they were about to have surgery?’ In response to this question, patients may have reported the duration of problems of their most recent episode with a specific level of severity rather than their overall duration [29]. Previous studies have reported that this may be due to the lack of clarity of the question that was used to elicit information about symptom duration [29]. Nevertheless, increasing symptom duration has recently been reported to be a significant predictor of poorer outcome after surgery [30].

This study is the first to examine the relationship between chronic diseases and patient-reported pain, functional status and duration of joint problems immediately before surgery in a large representative sample of patients. One possible interpretation of our findings is that the severity of joint problems at the time of surgery represents delays in access to surgery, although our findings do not give an indication about the length of these delays. It can also be argued that our observation that patients with chronic disease report more severe joint problems than patients without may also be explained by information bias (e.g. patients with more severe joint problems are more likely to have other comorbidities recorded) or that pre-operative conservative treatment may be more effective in patients without than in patients with comorbidities.

We acknowledge that our finding of an association between the presence of chronic conditions and more severe joint problems does not prove a causal relationship. However, our interpretation is supported by the findings from our previous qualitative study which explored the views of healthcare professionals on the referral and selection of patients with chronic diseases for hip or knee replacement surgery in the English NHS [11]. This study showed that chronic diseases may create subtle barriers, for example when patients who are considered to be unprepared for surgery are referred back to their general practitioner in primary care.

Furthermore, delays in access in patients with chronic conditions may be linked to patients’ reluctance to undergo surgery [31] or to clinicians’ uncertainty about the indications for replacement surgery [32–34]. In previous studies with different groups of healthcare professionals, the presence of chronic diseases was found reported to be a reason that surgery should not be recommended because of the increased risks of surgery [35]. This is also one of the arguments to justify the restricted eligibility criteria for hip and knee replacement imposed by some regional commissioners of healthcare in England who require that chronic diseases are optimised before surgery [6–8].

Delays to surgery have also been linked to patient health-seeking behaviour and reluctance to undergo surgery [36]. Firstly, differences in thresholds for pain may explain variation in seeking clinical advice or having surgery. There is evidence that people from more socio-economically deprived backgrounds – who are also more likely to have chronic diseases [37] – tend to accept a higher threshold of chronic pain and functional limitation before having surgery [38]. Secondly, a number of studies have reported differences in patient preferences and expectations for joint replacement surgery. For example, it was shown that elderly people may prefer to delay surgery and manage the pain and the limited mobility rather than undergo a surgery with risks of complications [39]. This may be similar for patients with chronic diseases given their higher risks of short-term complications after surgery [10]. Thirdly, patients with chronic diseases may also prioritise the treatment of their chronic diseases rather than starting surgical treatment of their joint problems [40].

There were several study limitations. First, it is important to mention in this context is that we could not adjust for the severity of the chronic diseases because the administrative data could capture the presence of chronic conditions but not their severity. This is especially important for the interpretation of the observed association between of the number of chronic conditions and the severity of the joint problems at the time of surgery. It is likely that we would have found an even stronger association if we had been able to take severity of the chronic conditions into account.

Furthermore, it is likely that there is a ‘healthy-surgical patient effect’. This effect may have contributed to an underestimation of the effect of chronic diseases as a consequence of the selection of patients for surgery being influenced by ‘unmeasured’ or ‘unobserved’ confounders that are not accounted for. As a result, patients with chronic diseases who had surgery may be less frail and less severe than patients with a similar chronic disease profile in the general population [41]. In addition, due to the lack of a control group (patients with comorbidities who have not had joint replacement surgery) it is not possible to fully account for this selection bias.

Also, our sample of patients represents 71% of all patients who had a hip or knee replacement in the English NHS between 2009 and 2016. While the response rate to the PROMs survey is high, non-recruitment may have led to confounders being unevenly distributed between different groups of patients and hospitals [42]. This was especially apparent with only 3% of patients reported to be from a minority ethnic group, although this may be explained in part by the high percentage of missing data (> 10%). To account for this, we adjusted for clustering of outcomes within hospitals and for socioeconomic factors. This adjustment had a minimal impact on the findings.

The negative consequences of the presence of comorbidities before surgery also need to be interpreted in the context of the outcomes after joint replacement surgery. In our previous systematic review of 70 studies looking at 10 outcomes comparing patients with and without patients chronic diseases, we found that chronic diseases predominantly had an impact on the safety e.g. complications) but little impact on the effectiveness (e.g. functional and pain outcomes) after joint replacement surgery [10]. This suggests that patients with chronic diseases do not benefit significantly less from hip and knee replacement surgery.

## Conclusions

In conclusion, patients with chronic diseases undergoing hip or knee replacement surgery reported more severe pain and a poorer functional status immediately before surgery than patients without chronic diseases. These findings suggest that on average patients with chronic diseases have hip or knee replacement later in the course of their joint disease, likely as a result of delays in access to surgery.

## Supplementary information

**Additional file 1.** Breakdown of OHS and OKS by pain and functional status questions (P = pain, F = functional status).

## Data Availability

The data that support the findings of this study are available from NHS Digital but restrictions apply to the availability of these data, which were used under license for the current study, and so are not publicly available.

## Abbreviations

HES: Hospital Episode Statistics
ICD-10: International Classification of Diseases, 10th Revision
MID: Minimally Important Difference
NICE: National Institute for Health and Care Excellence
OHS: Oxford Hip Score
OKS: Oxford Knee Score
PROMs: Patient Reported Outcome Measures programme.
