# Supplementary material for: Access to hip and knee replacement surgery in patients with chronic diseases according to patient-reported pain and functional status
Source: BMC Health Serv Res. 2020 Jul 1;20:602. doi: 10.1186/s12913-020-05464-3 (PMC7329455; doi:10.1186/s12913-020-05464-3)
Supplement: Supplementary file 1 — Additional file 1. Breakdown of OHS and OKS by pain and functional status questions (P = pain, F = functional status). [file 12913_2020_5464_MOESM1_ESM.docx]

Supplementary material – Breakdown of OHS and OKS by pain and functional status questions (P=pain, F=functional status)

|  | **Hip Replacement (OHS)** | **Knee Replacement (OKS)** |
| --- | --- | --- |
| **1** | How would you describe the pain you usually have in your hip? (P) | Describe the pain you usually have from your knee? (P) |
| **2** | Have you been troubled by pain from your hip in bed at night? (P) | How much trouble do you have washing and drying yourself? (F) |
| **3** | Have you had any sudden, severe pain-' shooting ', 'stabbing', or 'spasms' from your affected hip? (P) | How much trouble do you have getting in/out car or using public transport? (F) |
| **4** | Have you been limping when walking because of your hip? (F) | How long can you walk before pain becomes severe? (P) |
| **5** | For how long have you been able to walk before the pain in your hip becomes severe (with or without a walking aid)? (P) | After a meal how painful has it been to stand up from a chair? (P) |
| **6** | Have you been able to climb a flight of stairs? (F) | Have you been limping when walking? (F) |
| **7** | Have you been able to put on a pair of socks, stockings or tights? (F) | Could you kneel down and get up again? (F) |
| **8** | After a meal (sat at a table), how painful has it been for you to stand up from a chair because of your hip? (P) | Have you been troubled by pain in bed at night? (P) |
| **9** | Have you had any trouble getting in and out of a car or using public transportation because of your hip? (F) | How much has pain from your knee interfered with your normal work? (P) |
| **10** | Have you had any trouble with washing and drying yourself (all over) because of your hip? (F) | Have you felt your knee might suddenly give way or let you down? (F) |
| **11** | Could you do the household shopping on your own? (F) | Could you do the shopping on your own? (F) |
| **12** | How much has pain from your hip interfered with your usual work, including housework? (P) | Could you walk down a flight of stairs? (F) |
